# Juxtaglomerular apparatus hyperplasia under dual angiotensin blockade. A footprint of adequate RAS inhibition or a concern for renal fibrosis?

**DOI:** 10.1186/1471-2369-13-21

**Published:** 2012-04-25

**Authors:** Beatriz Fernandez-Fernandez, Alberto Ortiz, Carmen Gomez-Guerrero, Antonio Barat, Catalina Martín-Cleary, Jesús Egido

**Affiliations:** 1 Nephrology, IIS-Fundacion Jimenez-Diaz, Autonoma University Madrid, Spain; 2 Renal and Vascular Research Laboratory, IIS- Fundación Jimenez Díaz. Autonoma University Madrid, Madrid, Spain; 3 Pathology division , Fundación Jimenez- Díaz, Madrid, Spain; 4 IIS-Fundación Jiménez Díaz, Avda, Reyes Católicos 2, 28040, Madrid, Spain

**Keywords:** Angiotensin, Juxtaglomerular apparatus hyperplasia, Nephrotic syndrome, Paricalcitol, Renin

## Abstract

**Background:**

Dual renin-angiotensin system blockade with angiotensin-converting enzyme inhibitors and angiotensin receptor blockers has been advocated to minimize proteinuria. However, recent trials have questioned the renal safety of this approach. Our understanding on the molecular effects of dual blockade in humans is incomplete.

**Case presentation:**

We present a patient with corticoid resistant nephrotic syndrome who developed marked juxtaglomerular apparatus hyperplasia and renin expression in the context of dual angiotensin system blockade.

**Conclusions:**

Although renin may have profibrotic effects mediated by (pro)renin receptor activation, this report raises questions on the potential consequences of local renin activation on chronic kidney disease in patients with dual angiotensin blockade.

## Background

Angiotensin II blockade is the mainstay of antiproteinuric therapy [1,2]. Dual angiotensin system blockade with angiotensin-converting enzyme inhibitors (ACEIs) and angiotensin receptor blockers (ARBs) has been advocated to maximize the antiproteinuric effect [1,2]. However, the COOPERATE study was retracted and in the ONTARGET clinical trial dual blockade was more effective in decreasing albuminuria but increased the risk of adverse renal outcomes [3,4]. Diuretics enhance the antiproteinuric effect of RAS blockade with ACEi and ARBs [5,6]. More recently, a vitamin D receptor activator exerted a modest antiproteinuric effect as an add-on therapy in diabetic patients with angiotensin blockade [7]. Effective angiotensin II blockade may lead to a compensatory increase in renin production and juxtaglomerular apparatus hyperplasia in animal models, but there is little information in humans [8]. Calcineurin inhibitors have also been associated with juxtaglomerular apparatus hyperplasia attributed to chronic renal ischemia [9,10]. We present a persistently proteinuric patient who developed a remarkable juxtaglomerular apparatus hyperplasia in the course of dual angiotensin blockade for proteinuria. This case illustrates the limitations of currently available antiproteinuric approaches as well as the occurrence of physiological responses to therapy of unclear clinical significance.

## Case presentation

A 22-year-old male was referred to our hospital because of nephrotic syndrome. At age 17, in 2005, he was diagnosed of cranial cavernous sinus in the context of nephrotic syndrome. He was homozygous for the C677T polymorphism of the methylene tetrahydrofolate reductase (MTHFR) gene and had circulating lupus anticoagulant, being treated with acenocumarol, vitamin B6 and vitamin B12.

At diagnosis, blood pressure was 140/85 mmHg, serum creatinine (sCr) 1.6 mg/dl, proteinuria 8 g/day, serum albumin 1.5 g/dl, creatinine clearance 66 ml/min (24 hour urine collection), 25OH Vitamin D 11.2 ng/ml (normal 20–50) and calcitriol 21.7 pg/ml (normal 25–65). The genetic study was negative for podocin and nephrin gene mutations. The first renal biopsy performed in January 2006 showed focal segmental glomerulosclerosis, foci of tubular atrophy, mild interstitial inflammation and fibrosis, normal arterioles and small vessels and faint focal mesangial IgM deposits by immunofluorescence, with no abnormalities in juxtaglomerular apparatus morphology.

**Figure 1:**
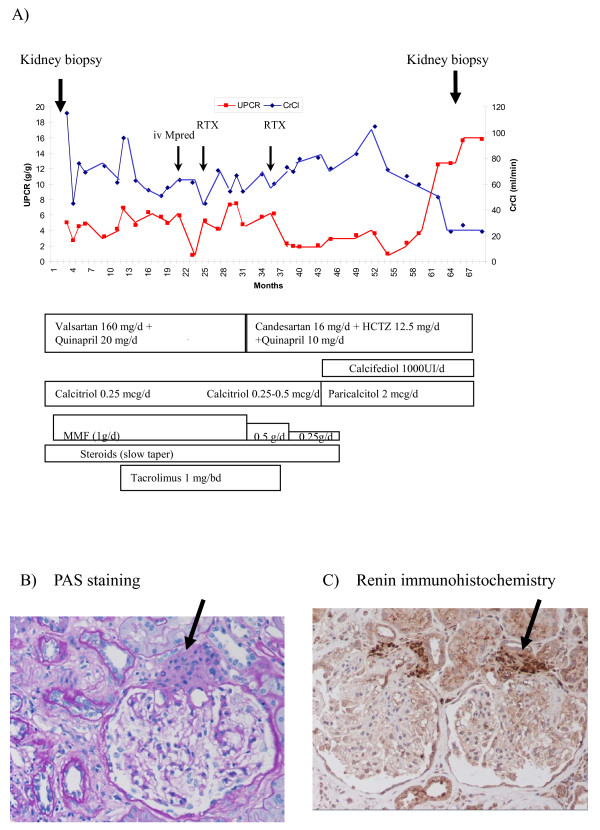
**Clinical course and renal biopsy findings.A**) Time-course of urinary protein/creatinine ratio (UPCR) and creatinine clearance (CrCl). Renal biopsies and prescribed therapies are indicated. **B**) Juxtaglomerular apparatus hyperplasia, PAS staining. **C**) Renin immunohistochemistry. Original magnification x200. The arrow indicates the juxtaglomerular apparatus.

He was treated with prednisone (starting at 1 mg/kg/day with slow taper) and mycophenolate mofetil (1-2 g/day) for two years. Nephrotic syndrome persisted (Figure 1.A). A short course of iv methylprednisolone (total dose 1250 mg) was followed by partial remission. Tacrolimus (1 mg/bd) was prescribed for 26 months and two cycles of Rituximab (700 mg per cycle) were added, the second of which was also followed by partial remission. Hypertension was initially treated with dual blockade (valsartan 160 mg/day plus quinapril 10 mg/day for three years, and then candesartan/hydrochlorotiazide 16 mg/12.5 mg plus quinapril 10 mg/day for two years) with ideal blood pressure control (120-124/70-74 mmHg). Very low levels of serum calcium, even with clinical symptoms, and vitamin D deficiency were treated with calcium and calcitriol 2.5 μg/week but not with native vitamin D. In June 2009, calcitriol was replaced by 25-hydroxy-vitamin D3 (2000 units/week) plus paricalcitol (1–2 μg/day). Complete remission was not achieved. In May 2010 a second renal biopsy was performed, containing 13 glomeruli: 4 with periglomerular fibrosis and partial retraction of the glomerular tuft, 3 sclerosed and 2 glomeruli with focal segmental lesions. A remarkable juxtaglomerular apparatus hyperplasia was observed in 4 glomeruli. (Figure 1.B). Interstitial fibrosis was present. Immunohistochemistry revealed granules inside the juxtaglomerular apparatus with intense renin staining (Figure 1.C). Unfortunately, plasma renin activity and serum and urine aldosterone levels at the time of biopsy were not available.

## Discussion and conclusions

The juxtaglomerular apparatus is located in the vascular pole of the glomerulus. Its main functions are the secretion of renin and direct control of glomerular haemodynamics via tubuloglomerular feedback. Renin-containing cells are differentiated afferent arteriole cells which contain (pro)renin packed into protogranules. Some granules will be secreted via the constitutive pathway releasing (pro)renin, others will be combined to form dense core vesicles that will convert (pro)renin into active renin. Renin release is the rate-limiting step of the renin-angiotensin system activation. Potential causes of juxtaglomerular apparatus hyperplasia include conditions that chronically stimulate renin production:

· Bartter and Gitelman syndromes

· Insulin-dependent diabetes mellitus with microalbuminuria

· Chronic renal ischemia

· Hypertrophy of the arterial and arteriolar walls

· Chronic volume depletion

· Chronic calcineurin inhibitor treatment, especially if nephrotoxicity is present

· ACEIs or ARBs

Most reports of juxtaglomerular apparatus hyperplasia correspond to patients with Bartter or Gitelman syndromes. Juxtaglomerular apparatus hyperplasia was also described in a female with chronic hypotension [11]. Mild enlargement of the juxtaglomerular apparatus has been reported in insulin-dependent diabetes mellitus patients with microalbuminuria [12]. Among iatrogenic causes we find chronic volume depletion following treatment with diuretics, chronic calcineurin inhibitor toxicity and treatment with ACEIs or ARBs [8,10,12].

In experimental animals prolonged treatment with toxic doses of cyclosporin A leads to interstitial fibrosis and tubular atrophy with mild hyperplasia of the juxtaglomerular apparatus that has been attributed to ischemia [12]. A non-significant trend towards an increased prevalence of juxtaglomerular apparatus hyperplasia was recently reported in patients with evidence of calcineurin inhibitor toxicity when compared to patients on calcineurin inhibitors without evidence of toxicity, although no information on angiotensin targeting therapy was provided [10]. Thus, it is unlikely that tacrolimus, which was stopped 26 months before the renal biopsy, was a main contributor to juxtaglomerular apparatus hyperplasia in this patient.

In animal models, ACEIs can modestly increase the juxtaglomerular apparatus size [8]. Less information is available in human subjects. Severe renal lesions, including juxtaglomerular apparatus hyperplasia and intense renin staining were present in twins from a pregnant woman treated with ARB (losartan) during pregnancy [13]. In our patient double angiotensin II blockade and diuretics might have contributed to the marked juxtaglomerular apparatus hyperplasia. In this regard, an effective angiotensin II blockade may be expected to increase compensatory renin production. In the past this might have been considered of scarce clinical relevance, given that the renin-angiotensin system was blocked downstream. However, current clinical evidence for potential adverse effects on renal function of dual angiotensin blockade [4] raise questions as to the potential molecular mechanism. In this regard, (pro)renin and renin may have direct profibrotic effects through activation of the (pro)renin receptor, raising concerns as to the consequences of juxtaglomerular apparatus hyperplasia [14]. Thus, activation of the (pro)renin receptor may promote angiotensin-independent intracellular signaling and secretion of the fibrogenic cytokine TGFβ1 [14]. Indeed a (pro)renin receptor blocker prevents diabetic nephropathy and cardiac fibrosis [14]. Following the renal biopsy dual angiotensin blockade was stopped and the renin-inhibitor aliskiren introduced in our patient. There is evidence that the magnitude of interstitial fibrosis may be attenuated to a greater degree by aliskiren than by an ACEIs, at least in experimental diabetic nephropathy [15]. Blood pressure was also under control but proteinuria remained unmodified. Persistent proteinuria may have contributed to the development of interstitial fibrosis (mean value during follow-up: 5.7 g/g creatinine).

Neither direct renin inhibitors nor drugs targeting aldosterone were used in this patient. However, the patient received paricalcitol at a dose that was recently reported to have additive antiproteinuric effects over ACEIs or ARBs in diabetic nephropathy [7]. Our understanding of the molecular mechanisms of kidney injury relies heavily on data from experimental animal models. Thus, there is ample evidence for the beneficial effect of vitamin D receptor activation in experimental kidney injury [16]‐[19]. Based on these experimental data it was hypothesized that paricalcitol inhibition of renin transcription was a key feature of its renoprotective effect [16]‐[19]. However, no differences in plasma renin activity were observed between paricalcitol- and placebo-treated patients in the VITAL study [7]. Our observation of juxtaglomerular apparatus hyperplasia and increased renin immunoreactivity despite years of full dose paricalcitol associated with 25OH vitamin D supplementation also argue against a key effect of vitamin D receptor activation on renin in humans. This does not preclude alternative mechanisms of a beneficial effect on paricalcitol on kidney injury, such as direct inhibition of the podocyte release of TGFβ1 and synthesis of extracellular matrix proteins [20] or an antiinflammatory effect independent of modulation of proteinuria [21].

In summary we observed a remarkable juxtaglomerular apparatus hyperplasia and increased renin expression in a focal segmental glomerulosclerosis patient on long term treatment with different immunosuppressive regimes, diuretics, and dual blockade with ACEI and ARB. However neither renin direct inhibitors nor aldosterone inhibitor agents were used. We hypothesize that despite vitamin D receptor activators, this therapeutic combination chronically stimulated the juxtaglomerular apparatus. This observation raises questions about a) potential adverse consequences of the current approaches to treat proteinuria through activation of compensatory mechanisms that have the potential to be injurious themselves and b) on the extrapolation of data on vitamin D regulation of renin obtained in experimental animals to humans.

## Consent

Written consent for publication was obtained from the patient.

## Competing interests

There is no competing interest with any financial organization regarding the material discussed in the manuscript.

## Authors’ contributors

Individual author contributions: BFF , AO and JE contributed substantially to the conception, design and writing of this case report. CGG was responsible for anti-renin staining and interpretation. AB interpreted the pathology samples. All authors read and approved the final manuscript.

## Pre-publication history

The pre-publication history for this paper can be accessed here:

http://www.biomedcentral.com/1471-2369/13/21/prepub
